# Safety and Biocompatibility of a Spray-Dried Influenza Microparticle Vaccine in Mice

**DOI:** 10.1007/s40883-025-00473-2

**Published:** 2025-09-15

**Authors:** A. C. Siddoway, D. Verhoeven, T. A. Harm, M. J. Wannemuehler, S. K. Mallapragada, B. Narasimhan

**Affiliations:** 1 Department of Chemical & Biological Engineering, Iowa State University, Ames, IA 50011 USA; 2 Department of Veterinary Pathology, Iowa State University, Ames, IA 50011 USA; 3 Department of Veterinary Microbiology & Preventive Medicine, Iowa State University, Ames, IA 50011 USA; 4 Nanovaccine Institute, Ames, IA 50011 USA

**Keywords:** Influenza, Vaccine, Biomaterials, Microparticles, Nanoparticles, Polymer

## Abstract

**Abstract:**

Influenza virus is a persistent source of morbidity and moribundity, and effective disease control requires ever-evolving effective vaccines. In this work, we evaluate the safety and biocompatibility of two novel polymeric particle-based influenza vaccines. Mice were immunized either intranasally or subcutaneously with these two formulations and examined at 1 h, 1 day, and 14 days post-immunization for histopathology in liver, kidneys, and lungs and serum biomarker analysis. Mice that received an intranasal vaccination were also observed for pulmonary disruption via whole body plethysmography. Examination of tissues post-immunization found only limited inflammation, with no difference observed in plethysmography measurements and no serum biomarkers (e.g., AST, AlkPhos) indicating tissue damage. Collectively, these data support the conclusion that these polymeric particle-based influenza vaccine formulations were well tolerated by the animals and did not induce any adverse side effects.

**Lay summary:**

Particle-based influenza vaccines were safety tolerated by mice and did not induce any adverse side effects.

**Supplementary Information:**

The online version contains supplementary material available at 10.1007/s40883-025-00473-2.

## Introduction

There is a pressing need to develop new and effective vaccine platforms against human respiratory pathogens such as influenza virus (IAV), SARS-CoV-2, and respiratory syncytial virus (RSV). In addition, the technical and logistical demands for preparing, storing, and administering such vaccines need to be considered as well. To this end, vaccines composed of polyanhydride microparticles prepared from 1,6-bis(*p*-carboxyphenoxy)hexane (CPH) and 1,8-bis(*p*-carboxyphenoxy)−3,6-dioxaoctane (CPTEG) as a 20:80 CPTEG:CPH copolymer and Pluronic F127®-based pentablock copolymer micelles provide multiple significant advantages over current influenza vaccines, which are either based on inactivated influenza virus (IIV) or live attenuated influenza virus (LAIV) approaches [1–3]. First, these biodegradable polymeric platforms are exceptionally biocompatible, with previous studies showing no material toxicity [4–6]. Second, polymeric particle systems can encapsulate and stabilize sensitive payloads and protect them from environmental impacts, such as temperature, both during vaccine storage and after vaccine administration, allowing for extended shelf life and sustained antigen release [7–9]. Third, sustained release of antigenic payloads enhances the magnitude and breadth of the resultant immune response, leading to the induction of robust humoral and T cell memory responses [10, 11]. Fourth, the sustained release kinetics can be tuned by changing chemical properties (i.e., molecular weight and copolymer composition of the biomaterial carrier) or enabling simultaneous delivery of multiple polymeric systems for ideal release kinetics [12, 13]. Fifth, small, hydrophobic polymeric particles are readily internalized via multiple phagocytotic and pinocytotic pathways, which benefit antigen processing and presentation by dendritic cells and macrophages [14–19]. This combined micro/nanoparticle approach allows for dose-sparing properties to be leveraged, leading to a more efficient use of expensive bioactive components [20]. Sixth, encapsulation within microparticles or association with micelles is a flexible process, allowing for a plug-and-play approach to rational vaccine design (e.g., through simultaneous encapsulation of multiple mRNA, proteins, and/or virions [21–27]). In addition, immunostimulatory compounds (e.g., TLR agonists, STING agonists.) may be included to further enhance and direct immune responses [28]. Seventh, tailored polyanhydride microparticles can be administered via multiple routes, either with a needle-free intranasal approach or traditional injection-based delivery based on the preferences of patients and providers, with profound implications for the immunocompromised and aged segments of the population [28–30]. Eighth, intranasal vaccination provides for the generation of local pulmonary CD4^+^ and CD8^+^ T cells along with the induction of germinal center B cells as well as systemic responses, in contrast to current vaccines that only generate systemic responses [30, 31]. Finally, the preparation of safer, more specific, less inflammatory, and more compliant vaccines (i.e., absence of preservatives, viral inactivating agents such as formalin, and needle-free delivery) may lower vaccine hesitancy [32, 33].

In this work, we build upon previous material biocompatibility and vaccine efficacy studies [4–6]—utilizing the same polyanhydride 20:80 CPTEG:CPH copolymer microparticles and Pluronic® F127-based pentablock copolymer micelles co-adjuvanted with either cyclic dinucleotides (c-di-guanosine monophosphate) or CpG-1668 ODN. While both polymeric systems act as depot forming delivery platforms, the pentablock copolymer micelles act like a surfactant solution. As these solutions are prone to foaming, they cannot be given intranasally because foaming will block air exchange. This limits the use of pentablock copolymer micelle solutions to subcutaneous administrations. Further, each polymeric system delivers payloads to different cellular locations. Polyanhydride particles are useful for endosomal delivery [34]—which enables delivery of CpG 1668 to endosomal TLR9. By comparison, pentablock copolymer micelles are effective at cytosolic delivery [35], and cytosolic STING agonists, like CDNs, are compatible with this platform. We further demonstrate the biocompatibility and safety of two influenza A vaccine formulations (i.e., containing multiple influenza antigens) based on these platforms, delivered intranasally (i.n.) or subcutaneously (s.c.). These polymeric particle formulations induced mild inflammation, necessary to initiate and sustain immune responses, without overt, tissue damaging inflammation. This mild (and transient) inflammation was observed in both histopathological tissue analysis and serum biomarker analysis.

Finally, we evaluated the efficacy of these polymeric particle-based vaccines in mice by challenging them with a mouse-adapted influenza virus and found that the subcutaneously administered formulation immune induced responses comparable to intramuscular IIV, while the intranasal formulation proved to be the superior vaccine. With these findings, this work sets a baseline upon which to base further polyanhydride microparticle- and pentablock copolymer micelle-based vaccine development in translational models and serves as a template for future applications with other respiratory pathogens, such as SARS-CoV-2 and RSV.

## Experimental Section

### Materials

Reagents for diacid and polymer synthesis are listed with supplier as follows: 1,6-dibromohexane, 1-methyl-2-pyrrolidinone, 4-*p*-fluorobenzonitrile, hydroxybenzoic acid, N,N-dimethylacetamide, N,N-(diethylamino)ethyl methacrylate (DEAEM), polyvinylpyrrolidone (MW = 40,000 Da), pyridine-2-carboxaldehyde, sebacic acid, and triethylene glycol were purchased from Sigma Aldrich (St. Louis, MO). Acetic acid, acetic anhydride, acetone, acetonitrile, alpha-bromoisobutyryl bromide, basic alumina powder, chloroform, copper acetate monohydrate, dimethyl formamide, ethyl ether, hexane, magnesium sulfate, methylene chloride, n-propylamine, pentane, petroleum ether, Pluronic® F127, potassium carbonate, sea sand, sodium borohydride, sodium hydroxide, sulfuric acid, triethylamine, and toluene were purchased from Fisher Scientific (Waltham, MA). Recombinant influenza A/HK/1/68 nucleoprotein (NP) was acquired from Sino Biological (Beijing, China). CpG ODN 1668 and cyclic dinucleotide (CDN) di-guanosine monophosphate were purchased from InvivoGen (San Diego, CA).

### Polymer Synthesis

CPTEG and CPH diacids were synthesized and used to prepare 20:80 CPTEG:CPH as previously described [36]. Briefly, the 20:80 CPTEG:CPH copolymer was synthesized by melt polycondensation by reacting appropriate molar amounts of CPTEG and CPH at 140 °C for 6 h under a vacuum of 0.1 torr [36]. The resulting copolymer was consistent with previous work in terms of molecular weight (8963 Da), purity, and molar composition (24:76), as measured via [1]H nuclear magnetic resonance spectroscopy (NMR) (Varian MR-500, 500 MHz, Varian, Palo Alto, CA) in deuterated chloroform [37]. Copolymer glass transition temperature (T_g_) was determined by differential scanning calorimetry (Q2000, TA Instruments, New Castle, DE) with a heating rate of 10 °C/min and a T_g_ of 29 °C for the 20:80 CPTEG:CPH copolymer was obtained.

Pentablock copolymers (PBC) based on Pluronic® F127 and DEAEM (PDEAEM_5_-PEO_100_-PPO_65_-PEO_100_-PDEAEM_5_) were synthesized via one-pot macroinitiation and atom transfer radical polymerization as previously reported [13, 38]. Briefly, Pluronic® F127 was converted to a macroinitiator by reaction with bromoisobutyryl bromide in toluene overnight. The resulting macroinitiator was used directly without purification. Addition of diethylamino ethyl methacrylate groups utilized atom transfer radical polymerization and was carried out under inert atmosphere at 70 °C for 20 h. The final polymer was obtained by passing the crude reaction mixture through an alumina column with a 50:50 methylene chloride:toluene mobile phase and precipitation of the final product in pentane. The molecular weight, purity, and composition of PDEAEM-Pluronic® F127-PDEAEM PBC were determined using ^1^H NMR in deuterated chloroform and ACQUITY Advanced Polymer Chromatography System (APC) (Waters Corporation, Milford, MA) with a polystyrene standard. The average molecular weight was ca. 15,500 Da, as measured by ^1^H NMR and APC. The number average molecular weight was measured by APC and found to be ca. 12,200 Da, with a polydispersity index of 1.27. All measurements were comparable with previous work [37]. In vitro cellular cytotoxicity of the PBC micelles was determined via MTT assay and was found to be comparable with previous work [38].

### Recombinant Protein Production and Purification

Recombinant HA trimer (H3N8 rH3_3_), based on influenza virus A/equine/1/KY/91 strain, was expressed as previously described [8]. Briefly, H3N8 rH3_3_ was produced using a commercial baculovirus system (Mirus Bio, Madison, WI) and HiFive cells (Invitrogen, Carlsbad, CA). H3N8 rH3_3_ was isolated from the supernatant via a 0.45 μm filter and by collecting retentate from a 100 kDa filter stirred cell system (Millipore Sigma). The protein was then characterized by native and denaturing/reducing polyacrylamide gel electrophoresis using Oriole staining (Bio-Rad, Hercules, CA) with specificity confirmation by western blotting using serum from H3N8 IAV vaccinated horses, as previously reported [8].

### Polyanhydride Microparticle Synthesis and Characterization

20:80 CPTEG:CPH microparticles were prepared as previously described using a Büchi B-290 Mini Spray Dryer (Flawil, Switzerland) equipped with a two-fluid nozzle [23]. A suspension was prepared with 10 mg/mL 20:80 CPTEG:CPH in strictly anhydrous methylene chloride to which a mix of either 1 wt.% H3N8 rH3_3_, 1 wt.% A/HK/1/68 NP, and 2 wt.% CpG 1668 or 1 wt.% H3N8 rH3_3_ and 1 wt.% A/HK/1/68 NP was added and sonicated for 30 s. The resulting suspension was directly spray dried at a rate of 10 mL/min, aspirator 70, and nitrogen supplied at 30 psi. The resulting particles were collected and stored at − 20 °C in vacuum sealed containers over desiccant until use. Size and morphology were determined using scanning electron microscopy (FEI Quanta 250, FEI, Hillsboro, OR). Particle zeta potential was measured using Zetasizer Nano ZS (Malvern, Southborough, MA).

Antigen release kinetics were determined as previously described [8]. Briefly summarizing, three different mixtures (*n* = 3 per mixture) were prepared: (1) ~ 10 mg of polyanhydride particles in 300 μL of phosphate buffered saline (PBS, pH 7.2); (2) 300 µL of a 100 mg/mL pentablock copolymer solution in PBS with 100 µg H3N8 A/equine/KY/91 HA and 100 µg H3N2 A/HK/68 NP; (3) ~ 10 mg of polyanhydride particles were suspended in 300 μL of 100 mg/mL pentablock copolymer solution in PBS with 100 µg H3N8 A/equine/KY/91 HA and 100 µg H3N2 A/HK/68 NP. All nine samples were incubated at 37 °C and were periodically centrifuged to pellet the particles, whereupon the supernatant PBS was removed and replaced with fresh PBS. The protein content of the supernatant PBS was determined using a micro-bicinchoninic acid assay (mBCA) (Pierce, Rockford, IL). Actual loading percent was determined similarly, with ~ 10 mg of microparticles incubated at 37 °C in 40 mM NaOH. Samples were collected every 24 h until the entire particle mass was degraded; total protein content was determined via mBCA and by summation of all release samples. Encapsulation efficiency was determined by comparing the actual loading with the theoretical loading, as described previously [8].

### Animal Vaccinations

**Fig. 1:**
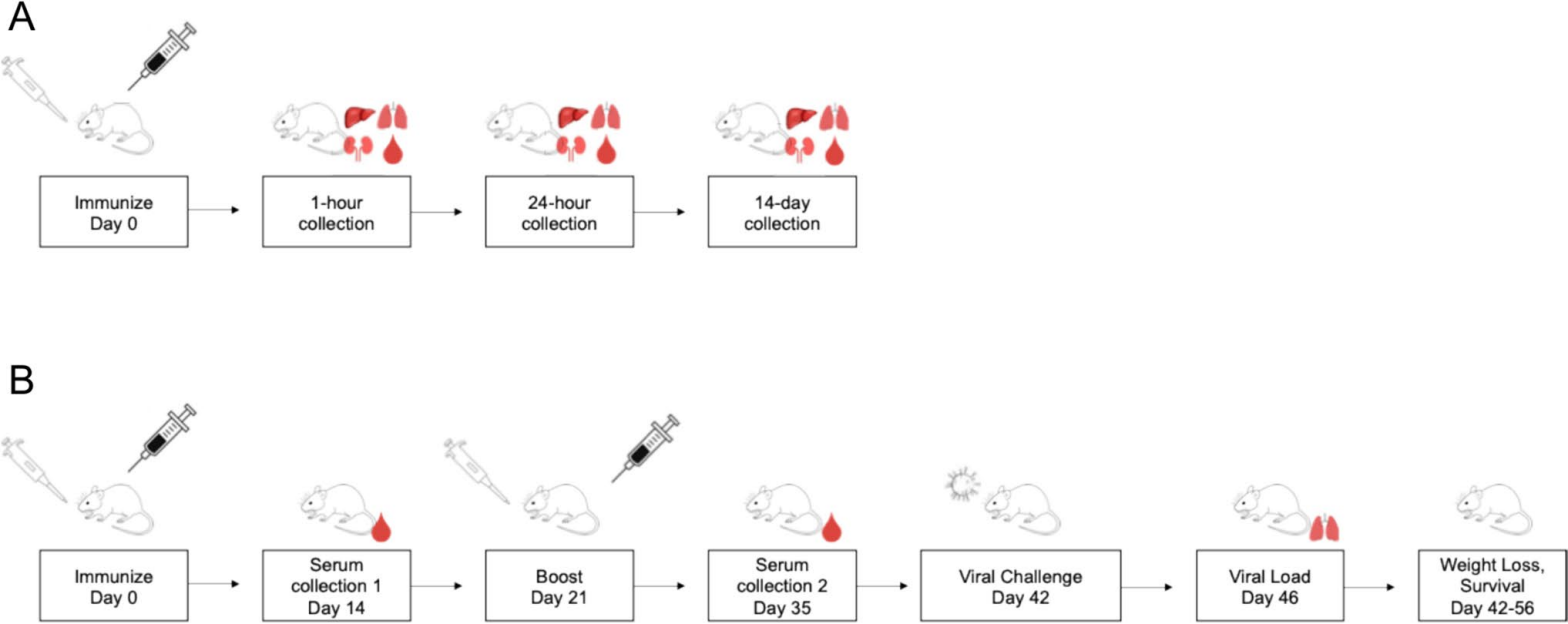
Experimental timelines for safety (**A**) and efficacy (**B**) studies. Sera, liver, kidney, and lungs (from intranasally vaccinated animals) were collected at each time point during the safety evaluation

Female C57BL/6 mice were purchased from Jackson Laboratories (Bar Harbor, ME). Animal procedures were conducted with the approval of the Iowa State University Institutional Animal Care and Use Committee. Two immunization studies were carried out to evaluate safety and efficacy, with vaccine groups summarized in Table 1 and study timelines shown in Fig. 1.

**Table 1:** Treatment groups

Vaccine group (per dose)	Particle mass (mg)	H3N8 rH3_3_ (μg)	A/HK/1/68 NP (μg)	CpG 1668 (μg)	CDN (di-GMP)	F127 PBC (mg)	Saline (μL)
Safety study groups							
Intranasal particle vaccine	5	10	10	10	–-	–-	50
Subcutaneous particle vaccine	5	10	10	–-	10	5	100
Antigen adsorbed on alum	–-	10	10	–-	–-	–-	25
Intranasal saline	–-	–-	–-	–-	–-	–-	50
Subcutaneous saline	–-	–-	–-	–-	–-	–-	100
Efficacy study groups							
Intranasal particle vaccine	5	10	10	10	–-	–-	50
Subcutaneous particle vaccine	5	10	10	–-	10	5	100
Inactivated influenza virus	–-	10*		–-	–-	–-	25
Naïve	–-	–-	–-	–-	–-	–-	–-

*Consisting of inactivated influenza A/HK/1/68 virus, normalized to contain 10 μg HA

In the safety studies, mice were euthanized at a predetermined time point following immunization. Whole blood, kidney, and liver samples were collected at all time points from all groups. Lung tissue samples and bronchoalveolar lavage fluid (see below) were also collected from the mice receiving the intranasal microparticle vaccine or intranasal saline.

For the efficacy studies, each group contained ten mice, four of which were euthanized at 4 days post-infection to assess viral load in the lungs. The remaining six mice were monitored for weight loss, lung function (penH), and survival.

### Plethysmography

Whole body plethysmography (WBP) (Buxco Electronics, St. Paul, MN) was used to measure enhanced pause (PenH) and minute-box volume (MVb). Baseline measurements were taken prior to intranasal vaccination and daily for 4 days afterward.

### Analysis of Serum Biomarkers

Blood was collected via cardiac puncture and centrifuged at 5000 rpm for 5 min at 4 °C to separate sera. Sera samples were stored overnight at 4 °C prior to analysis with Abaxis Comprehensive Profile (Zoetis, Union City, CA) at the Iowa State Clinical Pathology Laboratory. Results were compared with literature values provided by the manufacturer.

### Analysis of Bronchoalveolar Lavage Biomarkers

Bronchoalveolar lavage was collected at the time of necropsy. Mice were euthanized, and a sterile catheter was inserted into the exposed trachea of each mouse. One mL of sterile PBS was instilled into the lungs and then extracted while massaging the thorax of the mouse; this was repeated three times, with samples from each mouse combined. Samples were then centrifuged at 5000 rpm for 5 min at 4 °C and then stored at − 80 °C until analysis with a custom MILLIPLEX multiplex kit (MilliporeSigma, Darmstadt, Germany) targeting IP-10 (CXCL10), KC (CXCL1), MIG (CXCL9), IFN-γ, IL-10, TNF-α, MIP-2 (CXCL2), RANTES (CCL5), MCP-1 (CCL2), IL-6, IL-12, MIP-1a (CCL3), and IL-1β.

### Liver, Kidney, and Lung Histopathology

Whole organ tissue samples were collected and fixed in 10% neutral buffered formalin and transferred to 70% ethanol after 24 h. Liver and kidneys were collected from all groups, and lungs were also collected from the intranasal particle and intranasal saline groups. Sections from each organ were embedded in paraffin, sectioned at 5 µm thickness, and routinely stained in hematoxylin and eosin (H&E). H&E sections were evaluated, and photomicrographs were taken using an Olympus BX43 trinocular microscope equipped with a DP27 colored camera and CellSens Standard Software (Olympus Corporation, Japan). Tissues were blindly evaluated by a board-certified pathologist (TA Harm) using a histopathologic scoring system similar to those previously described [5]. The scoring system for the lung samples consisted of six independent scoring parameters. Parameters included inflammation, distribution of the inflammation, necrosis, BALT hyperplasia, edema, and hemorrhage. The scoring system for the liver and kidney samples consisted of six independent scoring parameters that included inflammation, distribution of inflammation, inflammatory distribution, necrosis, edema, hemorrhage, and extramedullary hematopoiesis (EMH). Each parameter was scored on a scale ranging from 0 to 5, and the sum of the scores was added per animal for a total possible score of 30. For inflammation, necrosis, edema, and hemorrhage, a score of 0 indicated no tissue affected, a score of 1 indicated 1–15% of the tissue was affected, a score of 2 indicated 16–30% of the tissue was affected, a score of 3 indicated that 31–45% of the tissue was affected, a score of 4 indicated that 46–60% of the tissue was affected, and a score of 5 indicated that greater than 60% of the tissue was affected. For inflammation distribution, a score of 0 indicated no lesion, a score of 1 indicated a focal lesion, a score of 2 indicated multifocal lesions, a score of 3 indicated coalescing lesions, a score of 4 indicated a locally extensive lesion, and a score of 5 indicated a diffuse lesion. For BALT hyperplasia, a score of 0 indicated no hyperplasia, a score of 1 indicated 2–3 hyperplastic BALTs, a score of 2 indicated 4–5 hyperplastic BALTs, a score of 3 indicated 6–10 hyperplastic BALTs, a score of 4 indicated 11–15 hyperplastic BALTs, and a score of 5 indicated greater than 15 hyperplastic BALTs. For EMH, a score of 0 indicated no EMH, a score of 1 indicated less than 5 EMH aggregates, a score of 2 indicated 6–10 aggregates, a score of 3 indicated 11–15 aggregates, a score of 4 indicated 15–20 aggregates, and a score of 5 indicated locally extensive infiltrates.

### Anti-H3N8 HA ELISA

**Fig. 2:**
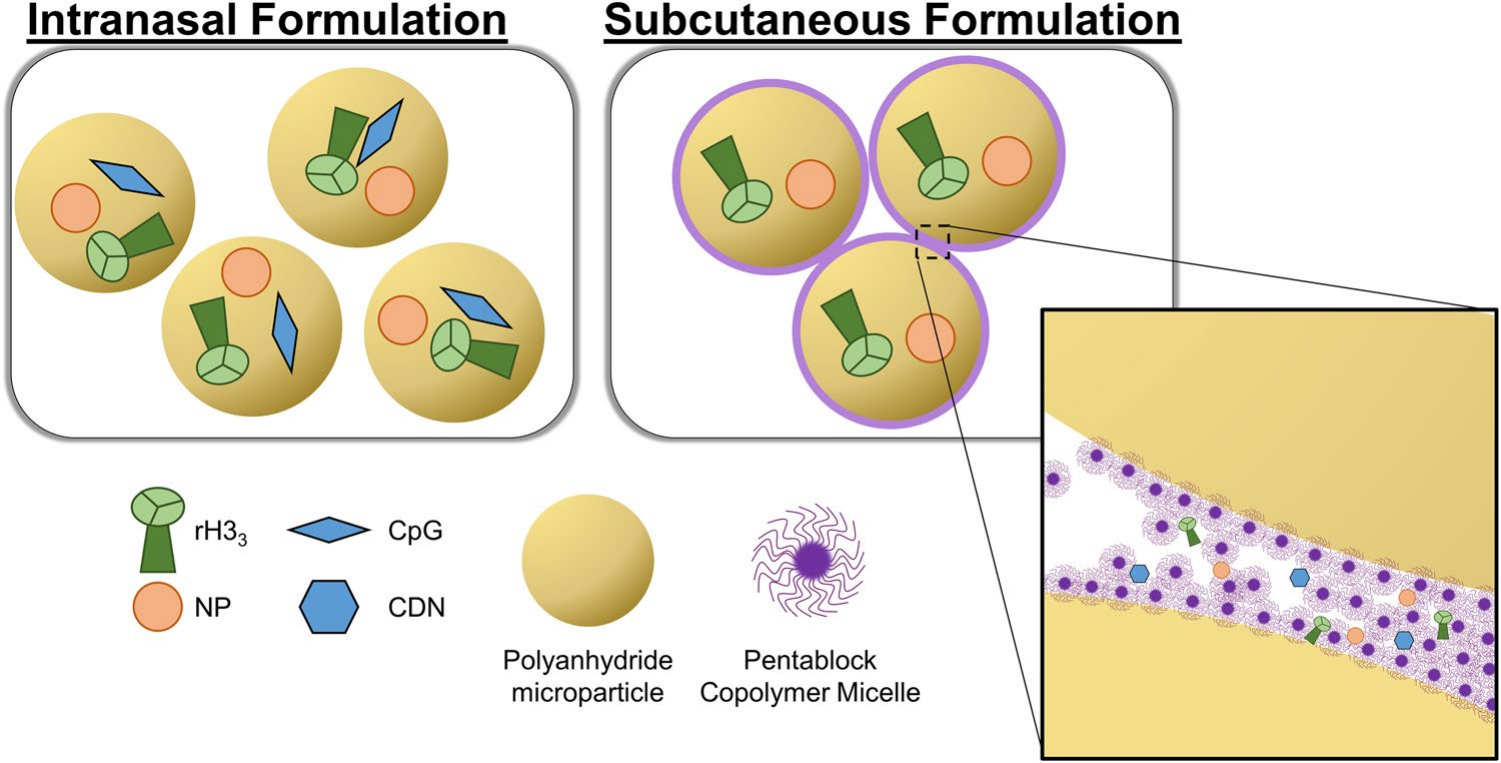
Schematic diagram of the intranasal formulation and the subcutaneous formulation. Particle scale is not to scale, with polyanhydride particles shown 20 × larger and actual size being 200 × larger

Anti-H3N8 HA antibody titers were measured using an enzyme-linked immunosorbent assay (ELISA) with horseradish peroxidase linked secondary antibodies. Blood was collected from the saphenous vein at 14 and 35 days (Fig. 2B), centrifuged at 5000 rcf for 15 min; sera were separated and stored overnight at 4 °C. High-binding, flat-bottom 96-well plates were coated with 100 μL 0.5 μg mL^−1^ H3N8 HA in pH 9.6 carbonate/bicarbonate buffer at 4 °C overnight. Wells were blocked with 1% (w/v) gelatin in PBS with 0.05% Tween-20 (PBS-T) for 2 h at room temperature. Plates were washed three times in PBS-T before adding 100 μL of PBS-T to each well. Sera samples were added to the first well in the microtiter plate at a dilution of 1:100; subsequently, two-fold serial dilutions were made across the plate and incubated at 4 °C overnight. Plates were again washed three times in PBS-T before adding 100 μL of PBS-T with secondary horseradish peroxidase-conjugated, goat anti-mouse IgG (H + L) at a dilution of 1:20,000. Plates were incubated for 2 h at room temperature and then washed four times in PBS-T followed by the addition of 75 μL ultra-TMB-ELISA substrate solution (Thermofisher, Waltham, MA). The reaction was allowed to proceed for 20 min and halted by the addition of 75 μL of 2 N sulfuric acid. The optical density (450 nm) was recorded using a plate reader (Spectramax, San Jose, CA). For analysis, the background is defined as the average optical density of the wells treated with sera from saline administered mice. Titer is defined as the reciprocal of the last dilution with an optical density greater than two times the background.

### Viral Challenge

For influenza challenge, mice were anesthetized with isoflurane to effect and challenged intranasally with an infectious dose of 15 LD_50_ of A/HK/1/68 (H3N2) in 25 μL sterile PBS (pH 7.6). The infectious viral dose for this viral culture in young, female C57BL/6 mice was determined previously in a challenge titration study (data not shown) finding 1 LD_50_ = 140 tissue culture infectious units (TCIU).

### Statistical Analyses

Statistical comparisons were performed using Graphpad (Prism 10, Graphpad Software, La Jolla, CA). Statistical comparisons of PenH and MVb measurements were performed using multiple nonparametric Mann–Whitney tests. Comparisons of serum biomarkers and bronchoalveolar lavage markers were performed using nonparametric Kruskal–Wallis and Mann–Whitney tests. Numerical data are reported as averages with the standard error of the mean. Comparisons of weight loss profiles after challenge utilized a one-way ANOVA with repeated measures, and survival curve comparisons were performed using Mantel-Cox log-rank tests comparing against naïve controls.

## Results

### Particle Characterization

Previous literature reports antigen structure preservation and delivery by polyanhydride-based particles and Pluronic®-based pentablock copolymer micelles [29, 37]. While previous studies utilized small batch synthetic methods, this work utilized spray drying—a scalable and industrially relevant method— to produce polyanhydride microparticles. The resulting microparticles were then delivered as an intranasal suspension or a subcutaneous suspension by combining with pentablock copolymer micelles (Fig. 2 and Table 2).

**Table 2:** Particle dynamic light scattering and zeta potential

Particles	Size (nm)	ζ potential (mV)
Polyanhydride microparticles	4385 ± 762	−23.3 ± 2.74
Pentablock copolymer micelles	20.6 ± 2.7	12.3 ± 1.85
Polyanhydride microparticles + pentablock copolymer micelles	4297 ± 561	12.7 ± 2.13

**Fig. 3:**
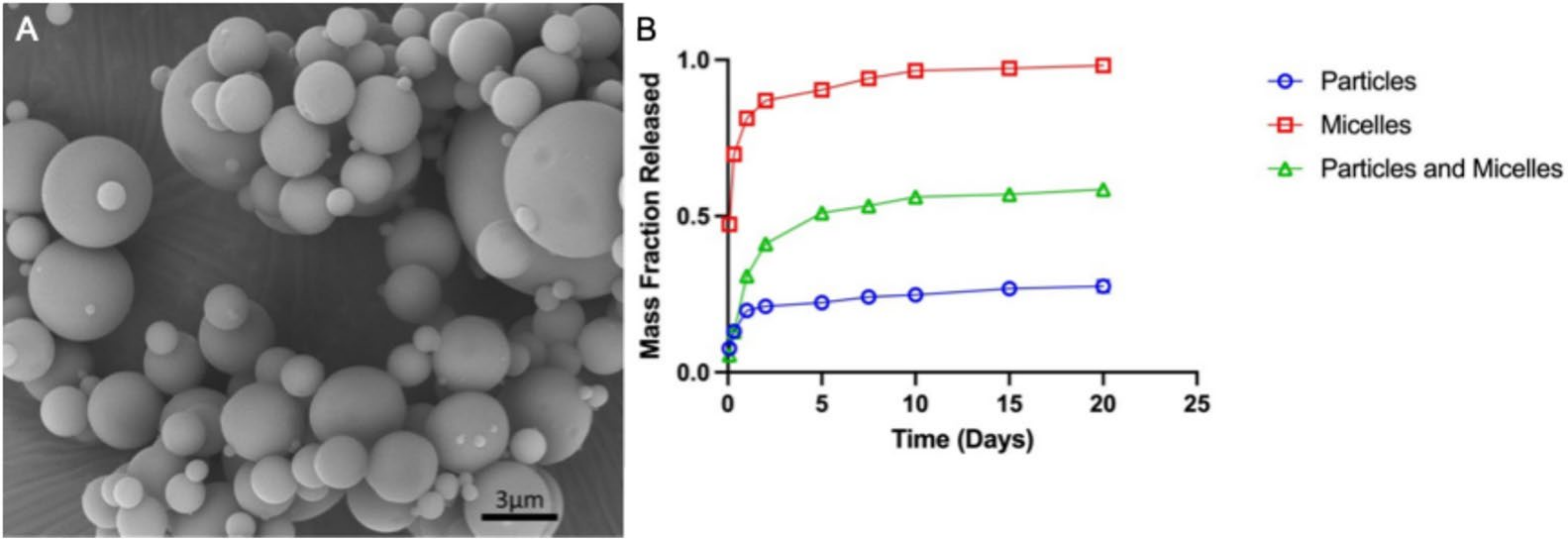
Scanning electron micrograph of the polyanhydride particles, scale bar represents 3 µm (**A**). Total antigen release kinetics from polyanhydride microparticles, pentablock copolymer micelles, and both polyanhydride microparticles and pentablock copolymer micelles. Error bars are smaller than symbol size, *n* = 3 per formulation (**B**)

Scanning electron microscopy was used to confirm the morphology of the polyanhydride particles (Fig. 3A). Total antigen release kinetics were monitored for 3 weeks, with antigen release from the pentablock copolymer micelles complete after 10 days (Fig. 3B). Polyanhydride microparticles exhibited sustained release throughout the observational window. Further characterization by DLS and zeta potential is summarized in Table 2. Consistent with previous work [8], polyanhydride microparticles are negatively charged and pentablock copolymer micelles are positively charged. The combination of both polymer systems results in an overall positive charge (Table 2), suggesting that the cationic micelles adsorb onto the surface of the anionic polyanhydride microparticles (Fig. 2). There is a slight increase in hydrodynamic size measured in the combination micelle and polyanhydride microparticle samples compared to the polyanhydride microparticles alone, further suggesting adsorption.

### Plethysmography

**Fig. 4:**
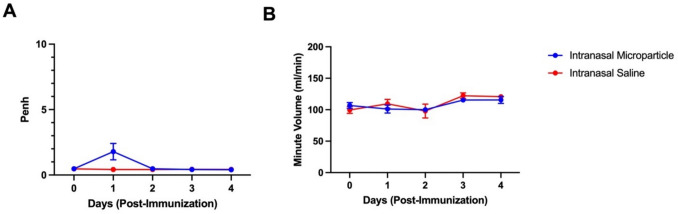
Intranasal plethysmograph measurements. Daily Penh (**A**) and MVb (**B**) measurements of mice immunized with the intranasal microparticle vaccine formulation or with an equivalent intranasal saline. Measurements are shown as mean ± SEM, *n* = 4. Statistical comparisons were done via nonparametric Mann–Whitney test, and no significant differences were observed. Differences 1 day post-immunization in the Penh plot (**A**) were trending towards significance, *p* = 0.065

In order to evaluate the impact of i.n. vaccination on airway function, animals were tracked via WBP for Penh and MVb (Fig. 4). Very small changes were observed 1 day post-immunization, but the Penh and MVb values for all the vaccinated mice returned to baseline measurements by day 2 post-immunization. At no time point was a statistically significant difference between the Penh and MVb values detected for the i.n. vaccinated mice. Furthermore, the observed increase in Penh observed at 24 h resolved by 48 h post-immunization (Fig. 4A).

### Serum Biomarkers

**Fig. 5:**
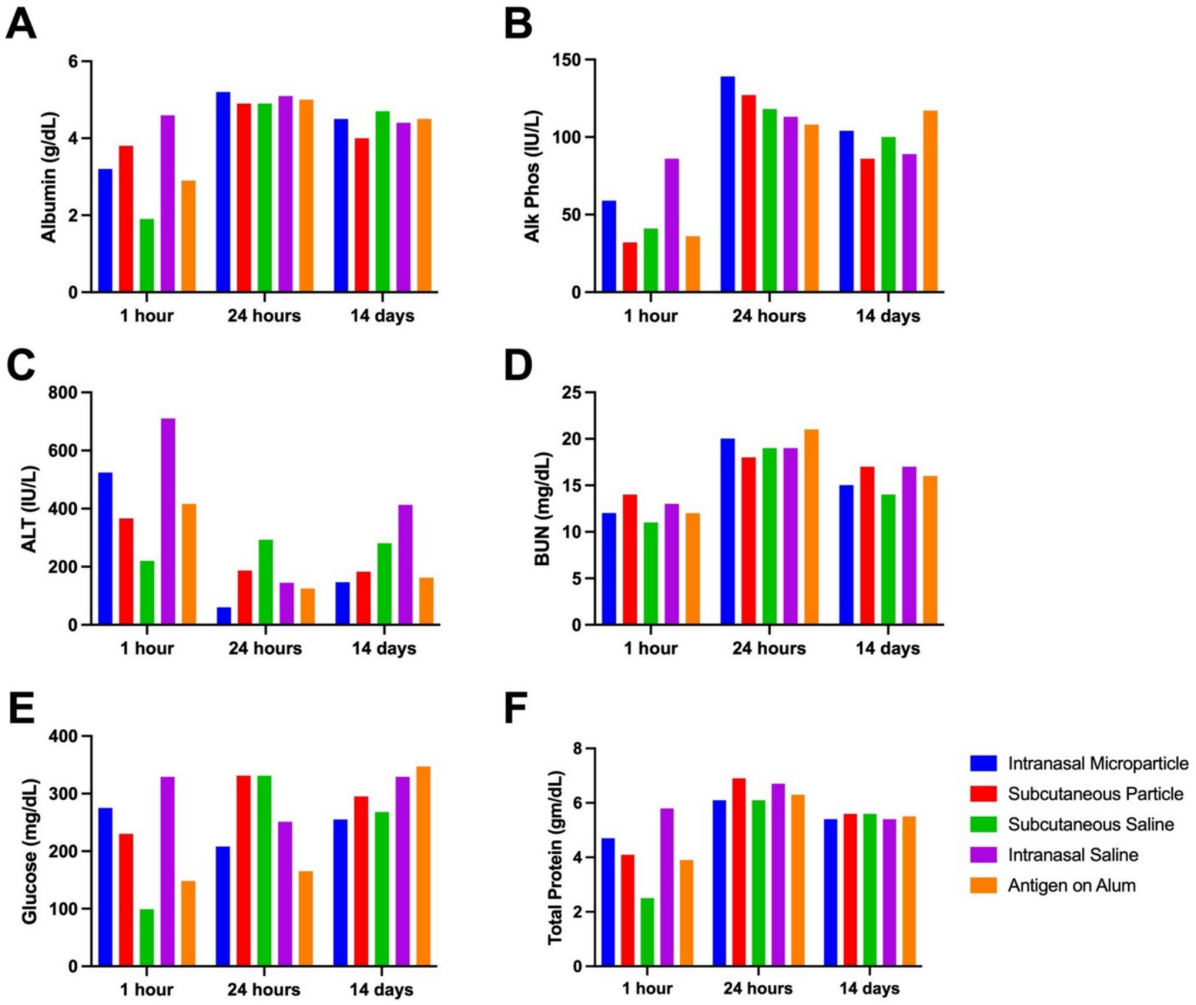
Serum biomarkers as measured by an Abaxis Comprehensive test at 1 h, 24 h, and 14 days post-immunization. Each bar represents sera collected from four mice at necropsy, pooled into one sample to meet test volume requirements, with dashed lines representing mean sera values in C57BL/6 mice as reported by Otto G, et al.[39] Albumin (**A**), alkaline phosphatase (**B**), alanine aminotransferase (**C**), blood urea nitrogen (**D**), glucose (**E**), and total protein (**F**) were measured. Perturbations of amylase, total bilirubin, calcium, phosphorus, creatine, sodium, and potassium from baseline saline animals were found to be negligible and are not shown

Following immunization, mice were euthanized at 1 h, 24 h, and 14 days post-immunization for tissue collection and evaluation of serum biomarkers to assess vaccine safety. Because of the volumes required, serum samples for each treatment group were pooled for analysis via Abaxis Comprehensive Profile and compared with Abaxis ranges and literature values [39]. Consistent with the plethysmography results in Fig. 4, insignificant differences in albumin, alkaline phosphatase, blood urea nitrogen, glucose, and total protein were observed (Fig. 5). At all three time points, the serum biomarker values for the vaccinated mice (regardless of vaccine formulation) were comparable to that of saline control mice at the respective time point. Calcium, creatine, phosphorous, and sodium levels were also unperturbed across all groups (and timepoints), and these values are not shown.

### Bronchoalveolar Lavage

**Fig. 6:**
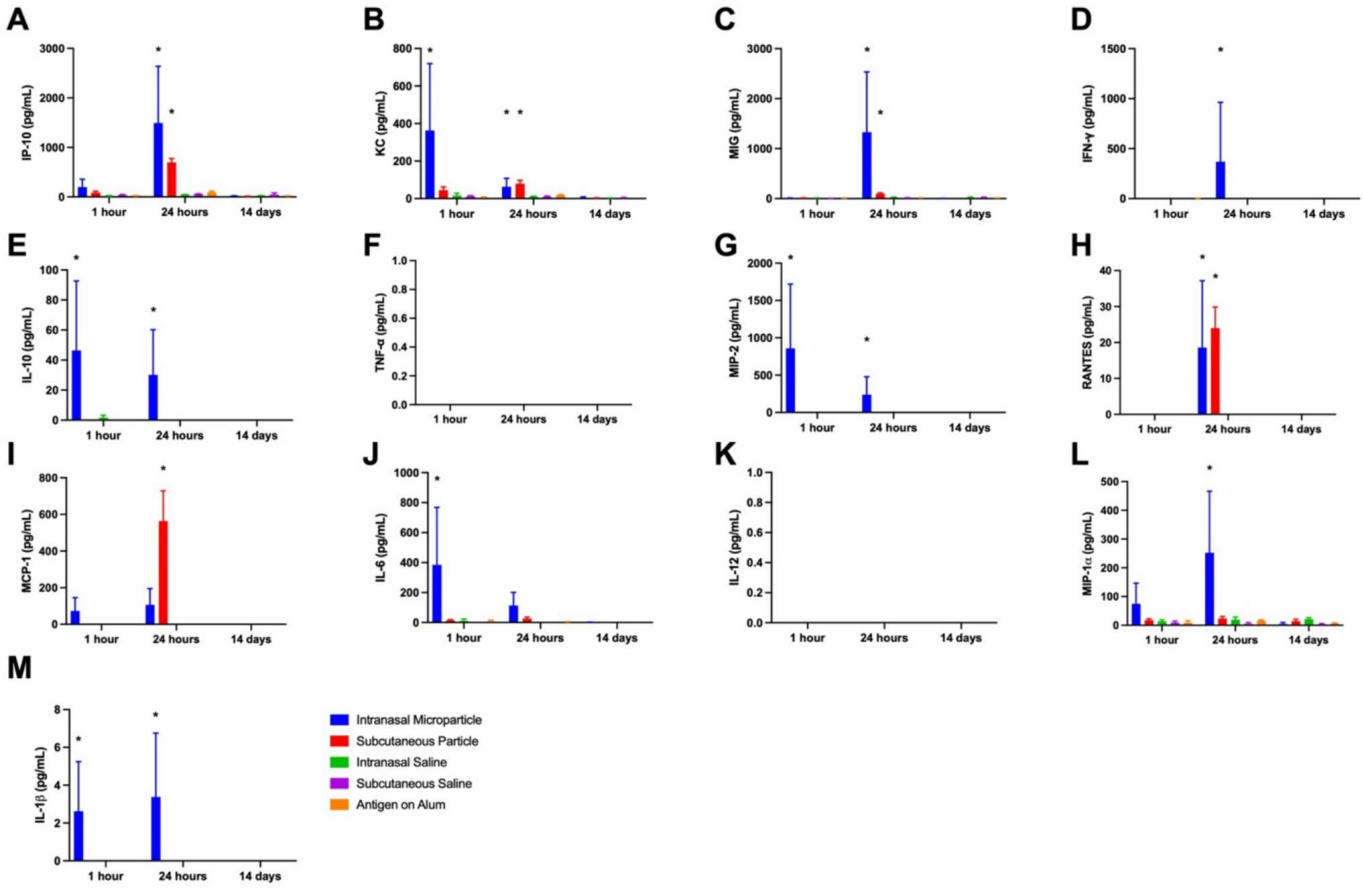
Measurement of cytokines and chemokines in the BAL fluid following intranasal administration of the particle-based vaccine. MILLIPLEX kit measuring IP-10 (**A**), KC (**B**), MIG (**C**), IFN-γ (**D**), IL-10 (**E**), TNF-α (**F**), MIP-2 (**G**), RANTES (**H**), MCP-1 (**I**), IL-6 (**J**), IL-12 (**K**), MIP-1α (**L**), and IL-1β (**M**) in BAL fluid. Significant upregulation was observed in the BAL of animals receiving both the intranasal particle and subcutaneous particle formulations, with greater variability in the intranasal formulation. Statistical comparisons were made using nonparametric Kruskal–Wallis and Mann–Whitney tests. * indicates *p* < 0.05

Bronchoalveolar lavage (BAL) collected at necropsy was analyzed by a custom MILLIPLEX multiplex kit designed to measure proinflammatory cytokines (Fig. 6). No significant response was detected in any of the samples collected from animals that received saline or antigen on alum intramuscularly. Significant upregulation of KC, TNF-α, MIP-2, IL-6, and IL-1β was observed in the BAL of animals 1 h post-immunization with the intranasal microparticle vaccine. By 24 h post-immunization, significant increases were present for IP10, MIG (*p* = 0.0065); IFN-γ, TNF-α, RANTES, MIP-1a, and IL-1β were detected in the BAL fluid. No significant responses were observed 1 h post-immunization in the BAL of animals receiving the s.c. combination vaccine formulation. Significant increases in the amounts of RANTES and MCP-1 detected in the BAL fluid were observed 24 h post-immunization in BAL of animals receiving the s.c. combination vaccine formulation. All these responses waned over time, and no inflammatory cytokines or chemokines were detected at 14 days post-immunization following either the intranasal or subcutaneous administration of the particle formulations.

### Histopathology

**Fig. 7:**
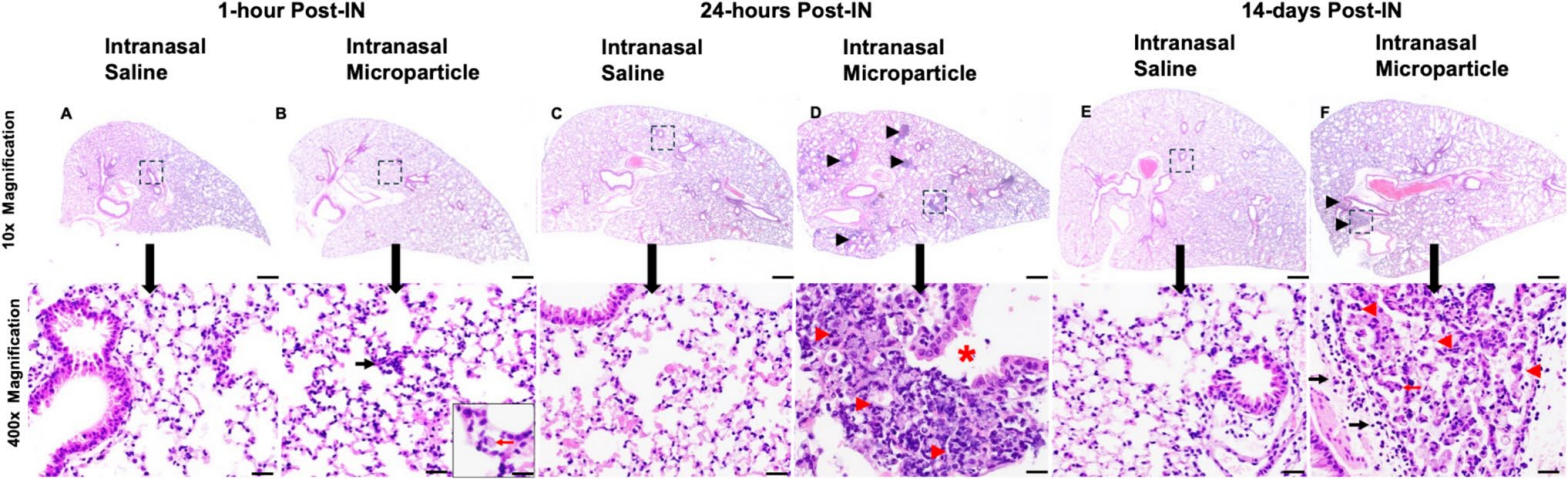
Lung tissue slides. Saline treated control lungs (**A**). Minimal intra-alveolar inflammation (arrow) was observed 1 h after intranasal (IN) inoculation (**B**). This infiltrate was composed of few neutrophils (black arrow) and foamy macrophages (red arrow, 600 × inset). Increased numbers of foamy pulmonary alveolar macrophages were also observed (**B**, inset). Multifocal areas of inflammation (black arrowheads) were observed in the lungs 24 h after IN inoculation (**D**). The inflammation at the 24-h time point was observed within the alveolar (red arrowheads) and terminal bronchiolar (asterisk). The infiltrate was composed of neutrophils and macrophages mixed with necrotic cellular debris and fibrin. Minimal to moderate inflammation (black arrowheads) was observed in the alveolar lumens 14 days after IN inoculation (**F**). The inflammatory infiltrate (red arrowheads) was composed of predominately macrophages and fewer neutrophils and multinucleated giant cells (red arrows). A mild to moderate inflammatory infiltrate (black arrows) composed of macrophages, lymphocytes, and plasma cells was observed in the perivascular and peribronchiolar interstitium (arrow). No inflammation is observed in the lungs of mice inoculated IN with saline at the 1-h (**A**), 24-h (**C**), and 14-day (**E**) time points. Dashed line boxes indicate the areas where the 400 × magnification images were from in each of the corresponding 10 × magnification images. Scale bars = 500 μm (10 × image), 50 μm (400 × image), 20 μm (600 × inset)

Tissue sections from the lung, liver, and kidneys were blindly evaluated and scored to evaluate the effect of the vaccine dose on tissue function as shown in Fig. 7 and Supplemental Figs. 1 and 2, with results summarized in Tables 3 and 4. Tissue sections were examined by light microscopy, and observed changes were assigned scores from 0 to 5 for six different metrics, as described in the “Materials and methods” section. The scores were then summed and averaged by group and are presented as total scores ± SEM (maximum 30 points) (Table 3). No lesions were observed in the 12 lung samples from the mice administered saline. Statistical analysis of scores was performed by Mann–Whitney *t*-tests comparing treatment groups against the appropriate control time point and route.

**Table 3:** Summarized histopathology scores

	**1-h p.i**			**24-h p.i**			**14-days p.i**		
	Lung	Liver	Kidney	Lung	Liver	Kidney	Lung	Liver	Kidney
Intranasal microparticle	1.25 ± 0.63	1.50 ± 0.50	0	5.00 ± 1.22	1.75 ± 0.48	0	1.75 ± 0.85	1.75 ± 0.75	0
Subcutaneous microparticle	–-	0.50 ± 0.29	0	–-	0	0	–-	1.75 ± 0.48	0
Antigen on alum	–-	1.25 ± 0.63	0	–-	0.50 ± 0.29	0	–-	1.75 ± 0.75	0
Intranasal saline	0	1.00 ± 0.71	0	0	1.50 ± 0.50	0	0	2.25 ± 0.75	0
Subcutaneous saline	–-	2.00 ± 0.57	0	–-	1.00 ± 0.75	0	–-	1.00 ± 0.71	0

**Table 4:** Statistical analyses of histopathology scores

	**1-h p.i**			**24-h p.i**			**14-days p.i**		
	Lung	Liver	Kidney	Lung	Liver	Kidney	Lung	Liver	Kidney
Intranasal microparticle vs intranasal saline	0.1429	0.6286	> 0.9999	0.0286	0.4857	0.9999	0.1429	> 0.9999	> 0.9999
Subcutaneous microparticle vs subcutaneous saline	–-	0.1714	> 0.9999	–-	0.4286	> 0.9999	–-	0.4000	> 0.9999
Antigen on alum vs subcutaneous saline	–-	0.6571	> 0.9999	–-	0.6571	> 0.9999	–-	0.7429	> 0.9999

At 1 h post-administration, analysis of the lungs in animals treated with the intranasal microparticle vaccine found that 75% (3 out of 4 animals) received a minimal inflammatory score of 1 with a distribution of inflammation score of 2 (multifocal). The inflammatory infiltrate was characterized by low numbers of neutrophils within the terminal bronchiolar and alveolar lumens. Scattered, low numbers of foamy pulmonary alveolar macrophages were observed within the adjacent alveolar lumens.

At 24 h post-immunization, all the mice treated with the intranasal microparticle vaccine displayed mild inflammation, with half of the group showing multifocal to coalescing infiltration patterns. The inflammatory infiltrate in two mice with higher inflammatory scores (scored 3–4) was characterized by large numbers of neutrophils (viable and degenerate) mixed with cytolytic and karyorrhectic debris, edema, mild hemorrhage, and foamy macrophages within the alveolar and bronchiolar lumens. In these same mice, low numbers of macrophages, lymphocytes, and plasma cells mildly expanded the peribronchiolar interstitium. The inflammatory infiltrate in the two mice with lower inflammatory scores (scored 2) was characterized by low to moderate numbers of neutrophils mixed with scant polymerized fibrin and cellular debris. Two mice received a score for necrosis, which was assigned due to the mild attenuation of respiratory epithelium and karyorrhectic debris in areas of inflammation. The epithelial necrosis and karyorrhectic debris appeared to be a bystander effect.

At the final 14-day post-immunization timepoint, 75% of mice in the intranasal microparticle vaccine group received scores above 0. Two mice received an inflammatory score of 2 with a distribution score of 2 (multifocal), and one mouse received an inflammatory score of 1 with a distribution score of 1 (focal). Overall, the inflammatory changes were minimal to mild. The inflammatory infiltrate in all affected mice was characterized by low to moderate numbers of macrophages, few multinucleated giant cells, and few neutrophils within the alveolar and bronchiolar lumens. The perivasculature and peribronchiolar interstitium in areas of inflammation were mildly expanded by macrophages, lymphocytes, and plasma cells.

Liver and kidney samples were also examined microscopically to screen the organs for any evidence of systemic hepatotoxicity or renal toxicity. Liver and kidney samples from mice in all groups were evaluated. No differences were observed in the kidney samples, with all tissue samples receiving no score (0) in all categories. No significant differences (Table 4) were observed in the liver parameters between when the treatment groups were compared to the control groups. Mice from each group received scores for focal (score 1, mononuclear cells and few neutrophils) inflammation (1) and extramedullary hematopoiesis. Focal inflammation and extramedullary hematopoiesis were observed as background lesions in mice. These changes were interpreted as background (i.e., normal), as there was no statistical difference between the scores of particle- and saline-treated mice (Table 3). Histopathologic examination of liver and kidney tissue did not identify any changes consistent with toxicity, inflammation, or necrosis in response to treatment.

### Vaccine Efficacy

**Fig. 8:**
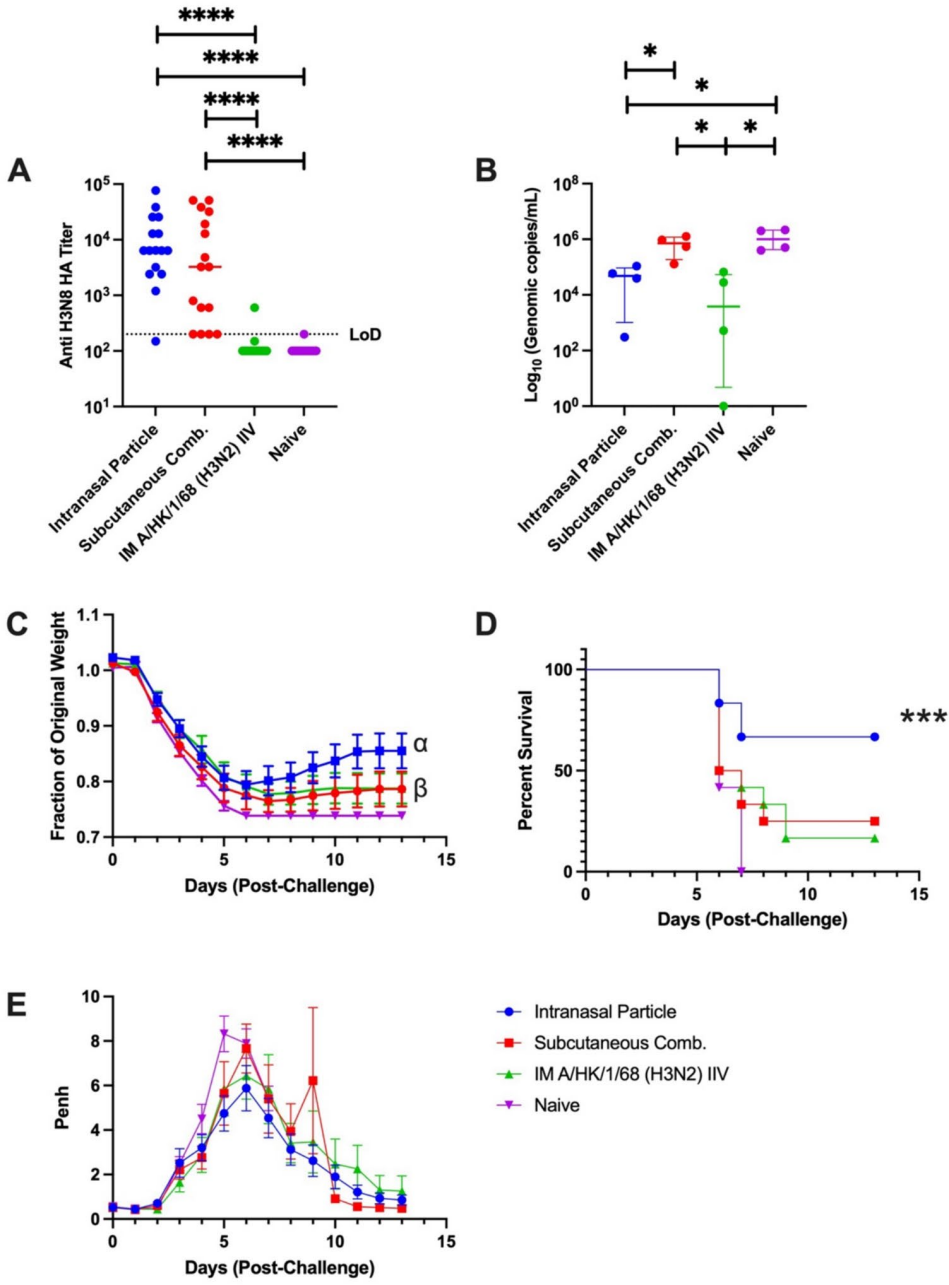
Vaccine efficacy following challenge with 15 LD_50_ of IAV (*n* = 16). Day 35 post-immunization anti-H3N8 HA total IgG titer (**A**). Viral titers as measured at 4 days after infection (**B**) (*n* = 4). Weight loss profile (**C**), survival (**D**), and Penh (**E**) throughout the duration of challenge (*n* = 12). Statistical differences in total IgG titer (**A**) and viral load (**B**) were determined via an initial check for significance by a Kruskal–Wallis test, with specific *P* values determined by Mann–Whitney* t*-tests. *P < 0.05, **P < 0.01, ***P < 0.001, ****P < 0.0001. Any unspecified comparison was found to be non-significant. Statistical differences in weight loss profiles (**C**) and Penh profiles (**E**) were determined via one-way ANOVA with repeated measures comparing against the naïve group. (*α*) denotes *P* < 0.0001 against the naïve group, and (*β*) denotes *P* = 0.0001 against the naïve group. Statistical differences in survival curves (**D**) were determined via Mantel-Cox log-rank test comparing against the naïve group

Mice were immunized using a prime-boost regimen (day 0 and day 21) with either an intranasal particle-based vaccine, a subcutaneous combination vaccine, intramuscular inactivated A/HK/1/68 (H3N2) virus vaccine, or left as naïve controls. Serum was collected at day 35 and analyzed via anti-H3N8 HA responses (Fig. 8A). While anti-H3N8 antibody titers in the sera from all the vaccinated groups were higher than that in the sera of naïve controls, we observed that the two particle-based vaccines induced significantly higher antibody titers than the IIV. Upon a highly stringent 15 LD_50_ challenge, mice that received the intranasal or intramuscular control vaccine had significantly reduced viral titers (Fig. 8B). Mice from all the vaccinated groups showed improved weight loss profiles over the naïve controls (Fig. 8C), but only mice that received the intranasal particle vaccine exhibited improved survival (Fig. D). All animals exhibited significant disruption to airway function (evidenced by the Penh profiles) regardless of vaccine group, presumably due to the high challenge dose administered (Fig. 8E).

## Discussion

Previous work from our laboratories has demonstrated the biomaterial safety and biocompatibility of 20:80 CPTEG:CPH polyanhydride microparticles and Pluronic F127®-based pentablock copolymer micelles [4, 40]. Later work demonstrated the efficacy of the vaccine formulations based on these same biomaterial systems against influenza A virus, RSV, and SARS-CoV-2 infections [29, 37, 41–43]. The current work aimed to build upon these previous studies by establishing the safety and biocompatibility of biomaterials-based influenza A virus vaccine formulations, which contain A/equine/1/KY/91 (H3N8) HA and A/HK/1/68 NP together with a route-specific co-adjuvant (Table 1).

As polyanhydride microparticles and pentablock copolymer micelles end up in different intracellular compartments [14, 16, 35], intranasal and subcutaneous combination vaccine formulations were designed considering particle destination (Fig. 1). Polyanhydride particles are generally internalized via endosomal compartments [14, 16], facilitating CpG 1668 delivery to endosomal TLR9 receptors. By comparison, pentablock copolymers are themselves adjuvants (i.e., facilitating crosslinking B cell receptors [44]) and are effective at facilitating cytosolic delivery. Cytosolic STING agonists, like cyclic-GMP CDNs, are appropriate adjuvants [35]. The final formulations shown in Fig. 1 depict an intranasal formulation that relies solely on polyanhydride particles to stabilize and deliver A/equine/1/KY/91 (H3N8) HA, A/HK/1/68 NP, and CpG 1668 as a mucosal adjuvant [45]. The subcutaneous combination formulation utilized polyanhydride particles to facilitate the long-term, sustained release profile, while also including soluble A/equine/1/KY/91 (H3N8) HA, A/HK/1/68 NP, and CDNs entrapped in pentablock copolymer micelles to provide antigen more rapidly to B cells.

While previous work has found these and similar formulations to be effective [29, 37], it is important to consider the safety and toxicological profiles of these vaccine formulations when administering them intranasally into the upper and lower respiratory tracts. The hydrophobic microparticles, with a slow but sustained release of H3N8 HA, H3N2 NP, and CpG 1668, are designed to recruit macrophages to the airway, similar to actual influenza A virus infection, but without the inflammation caused by the disease. The observed minimal disruptions to Penh and MVb measurements at 24 h post-immunization (Fig. 3) are consistent with the observed macrophage and neutrophil activity at 24 h post-immunization (Fig. 6), demonstrating mild inflammation but with sufficient cellular responses necessary for the induction and development of humoral and cell-mediated responses. Histopathological analysis at day 14 (Fig. 6) did not show evidence of persistent or chronic tissue damage (e.g., fibril formation) which is imperative in a repeat (i.e., prime–boost) regimen like vaccination.

Further evaluation of an inflammatory response was performed by analysis of serum biomarkers and cytokine/chemokine analysis of bronchoalveolar lavage (Figs. 2 and 3). Animals that received the intranasal microparticle vaccine had significantly more biomarkers present in their BAL than the animals that received a subcutaneous combination vaccine. This is not surprising given that the intranasal vaccine was contained within the lungs in contrast to subcutaneous fascia. The observed markers also generally matched previous work performed with primary cells collected from vaccinated animals, though the sensitivity of BAL fluid is significantly lower than that of primary cell supernatants utilized previously (Fig. 5) [28]. Importantly, regulatory and proinflammatory IP-10 (CXCL10) was detected alongside IL-6, which is necessary for primary and memory T cell responses and associated with polyanhydride particle-based vaccines [19]. Additional T cell-dependent markers, such as KC (CXCL1), MIG (CXCL9), IFN-γ, MIP-2 (CXCL2), RANTES (CCL5), MIP-1α (CCL3), and IL-1β, are secreted by pulmonary epithelial cells, alveolar septal cells, and alveolar macrophages in response to polyanhydride particles and released CpG-1668 [28, 37].

Responses detected in BAL from animals treated with the subcutaneous combination vaccine formulation were expectedly more muted because the subcutaneous vaccine is expected to “flow” along the dorsal subcutaneous fascia and drain to local brachial and axillary lymph nodes, rather than have any impact on the lungs. Despite this, inflammatory and T cell-recruiting cytokines (e.g., IFN-γ, RANTES, and MCP-1) were detected in BAL fluid (Fig. 5) upon subcutaneous vaccination with the combination vaccine formulation. It is likely that these cytokines originate from the site of injection and proximal draining lymph nodes. This may aid the development of a systemic response, as well as developing local responses detected by BAL, regardless of intranasal or subcutaneous administration, as the vaccine particles can spread systemically and mimic a chronic infection, as observed in our previous studies [46, 47].

Disruption of liver and kidney function is monitored by measuring serum biomarkers, such as alkaline phosphatase and blood urea nitrogen. This is particularly relevant when considering micro/nanoscale materials are degraded and that the degradation components would be cleared from the body via the excretory organs, such as the liver and kidneys. Deviation from serum values obtained from the saline control mice was only observed in alanine aminotransferase (Fig. 4c), which may be caused by immune activation and hemolysis and/or release of the CpG [48, 49]. Given that hemolysis was observed in most of the sera samples acquired in this study, increased alanine aminotransferase levels are likely partially due to hemolysis, though not completely, as inflammation markers were also detected in BAL samples [50, 51]. All the other observed biomarkers were within normal ranges (i.e., when compared to saline administration) and tracked with mice that received a saline injection/intranasal administration. These observations once again bolster previous work that found that these particle-based vaccine formulations do not induce significant tissue damage, either during or after administration, and are generally biocompatible [5, 40, 52].

These findings are further reinforced by histopathological analysis of liver and kidney samples taken from study animals, as shown in Fig. 6 and Supplemental Figs. 1 and 2. Analysis did not find any changes consistent with toxicity, inflammation, or necrosis (Tables 3 and 4). Furthermore, inflammation observed within liver tissues of the intranasal microparticle vaccine and the subcutaneous combination vaccine formulations was mild in severity and was interpreted as a background lesion. The observed biocompatibility is especially significant considering that the particles persist in the tissue and that polymer degradation components will continually traffic away from the administration site, further supporting the safety and biocompatibility of these vaccine formulations.

The efficacy of these vaccine formulations was also evaluated using a stringent 15LD_50_ viral challenge (Fig. 8). While only anti-H3N8 HA IgG titers were measured, it is clear from the weight loss, survival data, and along with viral load data that the intramuscularly administrated inactivated A/HK/1/68 (H3N2) virus vaccine (IIV) induced a modest level of protection. Similarly, the subcutaneous combination vaccine induced responses that were comparable to those induced by IIV (Fig. 8C and D). In contrast, mice immunized with the intranasal particle vaccine exhibited significantly (*p* ≤ 0.05) improved weight retention (Fig. 8C) as well as survival post-challenge (Fig. 8D). Based on viral genomic copies, viral load was only moderately reduced in the intranasal and intramuscular vaccinated groups (Fig. 8B). Additionally, there were no distinct differences in pulmonary function noted (Fig. 8E). It is likely that the use of a 15 LD_50_ challenge dose was highly aggressive, resulting in residual viral genomic copies and pulmonary inflammation. Despite the use of a lethal viral challenge dose (i.e., all the naïve mice succumbed to the challenge), intranasal immunization with the particle-based vaccine provided for 70% protection.

As part of the efficacy studies, only serum antibody responses were measured; in this regard, previous studies have shown that these polymeric platforms also induced potent T cell responses and demonstrated durable, long-term antibody responses [28, 43, 53]. Furthermore, flexible (needle-free) delivery options and the absence of unpopular vaccine components (e.g., aluminum hydroxides) may also help enhance vaccine uptake, making these vaccine platforms even more attractive.

## Conclusions

In this study, we administered and evaluated two influenza vaccine formulations utilizing polyanhydride copolymer microparticles and Pluronic F127®-based pentablock copolymer micelles and demonstrated the safety and biocompatibility of both formulations. While inflammatory markers and mild inflammation were observed transiently in the lungs, there were also no significant histopathological or serum biomarkers indicative of tissue damage from either intranasal or subcutaneous administration of these formulations. Collectively, both the intranasal microparticle vaccine and the subcutaneous combination vaccine formulations were found to be highly biocompatible and safe for in vivo use. Finally, the subcutaneous combination vaccine induced protective responses comparable to current inactivated influenza vaccines, while the intranasal microparticle vaccine induced a superior level of protection against a lethal IAV challenge.

## Supplementary Information

Below is the link to the electronic supplementary material.

Supplementary file 1 (DOCX 16.6 MB)

## Data Availability

Data may be available by contacting the corresponding author.
