# Supplementary material for: Safety and Biocompatibility of a Spray-Dried Influenza Microparticle Vaccine in Mice
Source: Regen Eng Transl Med. 2025 Sep 15;11(4):1052–66. doi: 10.1007/s40883-025-00473-2 (PMC12852260; doi:10.1007/s40883-025-00473-2)
Supplement: Supplementary file 1 — (DOCX 16.6 MB) [file 40883_2025_473_MOESM1_ESM.docx]

# Supplemental Figures

Supplemental Figure 1: No histopathologic changes were observed in the liver sections from animals in Ag on Alum (A-C), saline-treated intranasal (IN) (D-F), Intranasal particle treated IN (G-I), saline-treated subcutaneous (SC) (J-L), Subcutaneous combination treated (M-O) at any time point. Asterisk highlights a central vein in the liver images. Dashed line boxes indicate the areas were the 400x magnification images were from in each of the corresponding 10x magnification images. Scale bars= 500μm (10x image) and 50μm (400x image).

Supplemental Figure 2 No histopathologic changes were observed in the kidney sections from animals in Ag on Alum (A-C), saline-treated intranasal (IN) (D-F), Intranasal particle-treated IN (G-I), saline-treated subcutaneous (SC) (J-L), Subcutaneous combination treated (M-O) at any time point. Asterisk highlights a central vein in the liver images (A-C). Arrowheads highlights a glomerulus, and the arrow highlights the proximal convoluted tubules in the kidney images (D-F). Arrowheads highlights a glomerulus, and the arrow highlights the proximal convoluted tubules in the kidney images (D-F). Dashed line boxes indicate the areas were the 400x magnification images were from in each of the corresponding 10x magnification images. Scale bars= 500 µm (10x image) and 50µm (400x image).
